# Mindfulness in Unemployment and Sense of Coherence: A Systematic Review of Quality of Life and Indirect Evidence

**DOI:** 10.3390/healthcare14183024

**Published:** 2026-09-15

**Authors:** Elena Merlo-González, David Bermejo-Martínez, Rubén García-Fernández, Bibiana Trevissón-Redondo, Natalia Calvo-Ayuso, Enedina Quiroga-Sánchez

**Affiliations:** 1 Fundación Jiménez Díaz School of Nursing—Autonomous University of Madrid, 28040 Madrid, Spain; elena.merlo@salud.madrid.org; 2 Health Research Institute-Fundación Jiménez Díaz University Hospital, Autonomous University of Madrid (IIS-FJD, UAM), 28040 Madrid, Spain; 3 SALBIS Research Group, Department of Nursing and Physiotherapy, Universidad de León, 24007 León, Spain; 4 SALBIS Research Group, Department of Nursing and Physiotherapy, Campus de Ponferrada, Universidad de León, 24400 Ponferrada, Spainbtrer@unileon.es (B.T.-R.); ncala@unileon.es (N.C.-A.); equis@unileon.es (E.Q.-S.); 5 Nursing Research, Innovation and Development Centre of Lisbon (CIDNUR), Nursing School of Lisbon, 1600-190 Lisbon, Portugal; 6 CECAVIS, Instituto de Investigación Biosanitaria de León (IBIOLEÓN), 24071 León, Spain

**Keywords:** mindfulness, meditation, mental health, resilience, quality of life, salutogenesis, sense of coherence, unemployment, complementary therapies

## Abstract

**Highlights:**

**What are the main findings?**

- Direct evidence on mindfulness interventions in unemployed or economically inactive populations is limited and heterogeneous, with most studies assessing mental health or psychological distress rather than quality of life specifically.
- Evidence on sense of coherence was predominantly indirect, while intervention characteristics varied considerably in duration, content and delivery format.

**What are the implications of the main findings?**

- Current evidence is insufficient to establish the effectiveness of mindfulness interventions for improving quality of life or sense of coherence specifically in unemployed or economically inactive populations.
- Further research should directly target these populations and use clearly defined interventions, outcomes and follow-up periods to determine their potential health benefits.

**Abstract:**

**Background/Objectives**: Unemployment is associated with adverse physical and mental health outcomes and may negatively affect quality of life. This review aimed to synthesize evidence on the effects of mindfulness interventions on quality of life in unemployed or inactive populations and describe evidence on the relationship between mindfulness-related practices and sense of coherence (SOC), as well as intervention characteristics. **Methods**: A systematic review was conducted using PubMed, Scopus, Embase, CINAHL and Web of Science in February 2026, accepting all types of studies in English or Spanish, except systematic reviews and meta-analyses. Two search strategies identified studies addressing mindfulness, quality of life and unemployment or inactivity, and mindfulness-related practices and SOC. Study selection, data extraction and risk-of-bias assessment were conducted independently by two reviewers. The PRISMA 2020 was followed. It is registered in PROSPERO. **Results**: Fourteen studies were included. Direct evidence from unemployed or inactive populations was limited and heterogeneous, mainly assessing psychological distress or related outcomes rather than quality of life. Evidence concerning SOC was predominantly indirect and derived from other populations. Intervention characteristics varied in duration, content and delivery format, with digital tools frequently incorporated in studies involving unemployed populations. **Conclusions:** Current evidence is insufficient to establish the effectiveness of mindfulness interventions for improving quality of life or SOC specifically in unemployed or inactive populations. Indirect evidence suggests potential benefits, but differences in populations, interventions and study designs limit applicability. Further research directly targeting these populations, with clearly defined interventions, outcomes and follow-up periods, is warranted.

## 1. Introduction

Job dismissal and unemployment are important stressful life events that may have adverse consequences for physical and mental health. Several studies have described associations between these events and health problems, including anxiety, depression and other physical health conditions, which may ultimately contribute to poorer quality of life [1,2,3,4,5].

Different approaches can be adopted to address the health consequences associated with stressful life events and their consequences. The traditional pathogenic model focuses primarily on the factors associated with disease, whereas Antonovsky’s salutogenic model emphasizes the origin of health and well-being and the resources that help individuals cope with stressful circumstances [6,7]. Within this framework, “general resources of resistance (GRRs)”, such as social support, money, knowledge, self-esteem and healthy habits, may facilitate individuals’ ability to cope with life challenges. Antonovsky also introduced the concept of “sense of coherence (SOC)”, which reflects a person’s global orientation towards life as comprehensible, manageable and meaningful. These three components—comprehensibility of the event, the manageability of the event (that is, feeling able to address a situation) and the meaningfulness (feeling that life has meaning and that there are goals to achieve)—are central to SOC and are closely related to the availability and use of generalized resistance resources [6,7]. In this context, salutogenesis is considered the broader theoretical framework whereas sense of coherence (SOC) is considered a specific construct through which the salutogenic dimension is assessed. SOC can be assessed using the 29 items Life-Oriented Questionnaire or the Sense of Coherence Questionnaire or its shorter 13 items version [8].

Quality of life is another relevant outcome when considering the consequences of unemployment or inactive population and approaches aimed at promoting health and well-being. In 1994, the World Health Organization defined quality of life as “individuals’ perceptions of their position in life in the context of the culture and value systems in which they live and in relation to their goals, expectations, standards and concerns” [9]. A salutogenic perspective is consistent with the comprehensive approach of Primary Care, where health promotion and individualized care consider the physical, emotional, psychological and social dimensions of health and take into account individuals’ values, preferences, goals and experiences [10,11].

From this perspective, health-promotion interventions may seek to strengthen individuals’ knowledge, skills and resources for coping with stressful situations and maintaining well-being. Previous intervention research has explored salutogenic approaches in different populations. For example, an intervention aimed at promoting cardio-cerebrovascular health from a salutogenic perspective reported improvements in SOC alongside changes in physical activity, work stress and other health-related parameters [12]. These findings provide a rationale for considering salutogenic approaches in situations characterized by significant psychosocial stress, while their applicability to unemployed populations requires specific investigation.

Interventions involving unemployed individuals have traditionally focused mainly on employability, job training and job-search strategies. In contrast, fewer studies have specifically examined interventions aimed at promoting the health and well-being of unemployed individuals. This gap is relevant because unemployment may affect multiple dimensions of health and quality of life and may therefore require approaches that go beyond employment-related outcomes.

Some studies evaluate the effectiveness of different interventions that promote health and improve the quality of life in the general population [2,3,4]. Among these, relaxation techniques, including breathing, yoga and mindfulness, have gained increasing attention in recent years [13,14,15,16,17]. These findings provide a rationale for investigating whether such approaches may also be relevant to populations experiencing unemployment, although evidence from other populations cannot be assumed to demonstrate effectiveness in unemployed individuals.

Mindfulness has been investigated as a health-promoting approach in different populations, including its potential effects on psychological well-being, quality of life and sense of coherence [3,14,18,19]. Some studies have also explored the relationship between mindfulness-related practices and SOC, providing preliminary evidence of potential associations with psychological health and well-being [13,20].

Mindfulness, meditation and yoga are related but distinct approaches. Meditation generally involves practices aimed at cultivating attention and mental calmness [21], whereas mindfulness emphasizes awareness of present experiences and the regulation of responses to them [19]. Yoga may combine meditation and mindfulness-related practices with physical postures or controlled physical activity [22]. Despite these differences, these approaches may share some components related to attention, awareness and well-being [19,21,22]. Consequently, they have been investigated in different populations and intervention formats, but their effects cannot necessarily be considered equivalent.

Despite growing interest in mindfulness and related health-promoting approaches, evidence on their effects on quality of life among unemployed populations remains limited. In addition, the relationship between mindfulness-related practices and sense of coherence has been explored across different populations, but the available evidence is heterogeneous. To address these gaps, this study aimed to:

1. To synthesize the existing scientific evidence on the effects of mindfulness interventions on quality of life in unemployed or inactive populations;
2. To describe the available evidence on the relationship between mindfulness-based practices and sense of coherence (SOC) across the populations represented in the included studies.

As a secondary aim, the methodological characteristics of the mindfulness-based interventions included in the review are described.

## 2. Materials and Methods

A rigorous and systematic approach has been followed to ensure the quality and relevance of the data obtained for this research. The eligibility criteria, search strategy, sources of information, study selection and data extraction process are detailed below. The PRISMA 2020 checklist (Supplementary Materials) was used to develop this systematic review.

Given the two distinct objectives of the review, two separate search strategies were conducted and subsequently analyzed as two distinct bodies of evidence. The protocol registration reference at PROSPERO is CRD420251179584.

It was decided not to conduct a meta-analysis due to the great variability in the characteristics of the different types of studies containing different endpoints and different sample sizes to analyze different objectives, which are poorly related between studies.

### 2.1. Search Strategy and Sources of Information

The search strategy was designed to identify relevant studies in the following databases: PubMed, Scopus, Web of Science, Embase and CINAHL. The searches were conducted during February 2026 using two independent search strategies corresponding to the two aims of the review. Consequently, rather than using a single PICO question, two complementary review questions were established according to the population and outcome addressed by each search.

The first search addressed quality of life in unemployed or inactive populations, whereas the second search addressed sense of coherence (SOC) across the populations represented in the available literature. During the development and refinement of the second search strategy, terms related to unemployed or inactive populations were initially considered; however, these population terms did not yield any results and were therefore removed from the final search strategy in order to identify the available evidence on the relationship between mindfulness-based practices and SOC across populations. Thus, the population criterion differed between the two search strategies according to the specific objective and the results obtained during the development of the search strategy. This distinction was maintained throughout the study selection and synthesis.

For the first search, the following equation was used using the MeSh terms: (mindfulness OR meditation) AND ((Quality of Life) OR well-being OR welfare) AND (unemploy* OR jobless OR (inactive population)). This equation was discussed and adjusted to maximize relevant results with the different research team members. For the second search, the following equation was used: (mindfulness OR meditation) AND (salutogen* OR (sense of coherence)). Both search strategies were also adapted and searched using the corresponding DeCS terms in Spanish.

Inclusion and exclusion criteria were defined to limit the search results to those relevant articles that are published from January 2021 to December 2025, including all types of qualitative and quantitative studies that were not systematic reviews or meta-analyses, in order to cover a wide variety of approaches and methodologies.

For the first search equation, studies were required to include an unemployed or inactive population. For the second search, no specific population condition related to employment status was required, because the aim was to identify evidence on sense of coherence across the populations represented in the included studies.

Articles that include mindfulness, meditation, yoga, or other techniques that use breathing as a basis for meditation were eligible for inclusion.

Only articles published in English or Spanish were accepted.

### 2.2. Study Selection Process and Data Extraction

The study selection, data extraction and methodological quality assessment were independently performed by two reviewers. Disagreements arising at any stage were resolved through discussion and consensus. When consensus could not be reached, a third reviewer was consulted. The Mendeley bibliographic manager 1.19.5 was used to identify and remove duplicate records. No automation tools were used for study selection or data extraction, and the assessment of the eligible studies was conducted manually.

Randomized clinical trials were reported in accordance with the CONSORT statement, and observational studies (cohort, case–control, and cross-sectional) followed the STROBE guidelines. Methodological quality and risk of bias were appraised using the Joanna Briggs Institute (JBI) critical appraisal tools appropriate to each study design. The results of the methodological quality and risk-of-bias assessment were considered in the interpretation and synthesis of the findings.

## 3. Results

### 3.1. Selection of Studies

**Figure 1:**
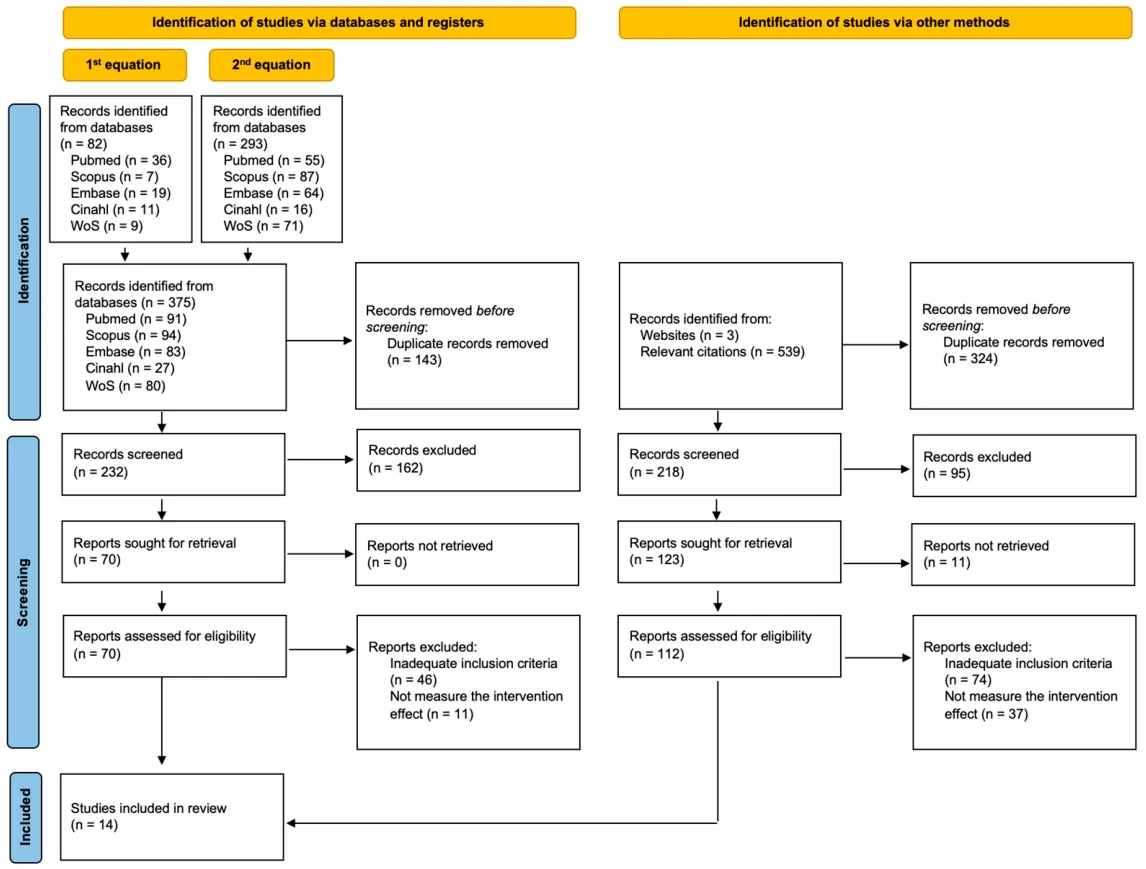
PRISMA 2020 flow diagram [23].

The two complementary search strategies were performed in PubMed, Scopus, Web of Science, Embase and CINAHL databases, identifying 375 records (Figure 1, made by the PRISMA Flow Diagram tool [23]). Of these, 82 were identified through the search addressing mindfulness or meditation in relation to quality of life and well-being among unemployed or inactive populations, and 293 through the search addressing mindfulness or meditation in relation to salutogenesis or sense of coherence. Following the screening and eligibility assessment process, 13 articles were included in the review.

The remaining articles did not meet the inclusion criteria by not assessing the effect of the intervention, not analyzing the intervention in the same study population, being systematic reviews or meta-analyses, or being written in a language other than English or Spanish.

An additional free search was conducted by screening the reference lists of the studies selected from the primary database searches and by following relevant citations identified during this process. A total of 542 additional records were screened using the same eligibility and exclusion criteria applied in the primary searches. After removal of 324 duplicates and exclusion of 95 records by title and abstract, 11 records could not be retrieved, and 112 records were assessed for eligibility. Finally, one additional study was included from the additional search in the review.

The 14 empirical studies included comprised three randomized controlled trials, five quasi-experimental studies, three analytical cross-sectional studies and two observational longitudinal or cohort studies. One qualitative study was also included. No case–control studies or clinical case reports were identified among the 14 included documents.

The studies were conducted in diverse geographical and population settings. The populations ranged from young unemployed adults and people affected by COVID-19-related unemployment to cancer patients, university and nursing students, psychotherapy patients, yoga or pilates practitioners and participants in an entrepreneurship training program. This heterogeneity is particularly relevant to the interpretation of the findings because the two evidence strands did not address the same population. Studies directly involving unemployed or economically inactive populations were therefore distinguished from studies providing indirect evidence regarding mindfulness, psychological well-being or sense of coherence.

The main characteristics of the included studies are presented in Appendix A.

### 3.2. Evidence in Unemployed and Economically Inactive Populations

Five studies provided the most direct evidence regarding populations affected by unemployment or job loss [24,25,26,27,28]. However, these studies differed substantially in their interventions, study designs and outcomes, and therefore their findings were not directly comparable.

Roemer et al. evaluated a low-dose mindfulness-based intervention among young unemployed adults and found that the intervention was associated with a reduction in psychological distress after accounting for baseline mindfulness and well-being. The authors reported that the intervention enhanced mindfulness and decreased psychological distress in their sample of young unemployed adults [24].

Other studies focused on employment-affected populations during the COVID-19 pandemic. Mata-Greve et al. examined unemployed or furloughed individuals together with essential workers, focusing on mental health and the perceived usability of digital mental health tools. The study found that, despite the potential need for these tools, their use remained limited and that the majority of participants who did not use a digital mental health tool had not considered seeking one to cope with the pandemic [25]. Comtois et al. similarly evaluated digital mental health interventions in a pragmatic randomized clinical trial involving unemployed individuals and essential workers, providing evidence from an employment-affected population but addressing mental health-related outcomes rather than quality of life [26].

Evidence from individuals who had experienced job loss was also provided by Marasigan. The study evaluated stress-management skills training using a quasi-experimental pretest–posttest design with experimental and control groups. Psychological well-being was assessed using the Ryff Psychological Well-Being Scale, and the experimental group showed improvement following the intervention [27].

Jiwani et al. provided observational evidence concerning employment status and psychological treatment outcomes in a large naturalistic psychotherapy sample. Rather than evaluating a mindfulness intervention, the study examined the relationship between employment status and symptoms, engagement and psychotherapy outcomes. Therefore, its findings provide complementary evidence regarding the psychological consequences associated with employment status rather than direct evidence of the effectiveness of mindfulness [28].

Taken together, the studies involving unemployed or employment-affected populations showed evidence of improvements in psychological distress or psychological well-being in some intervention contexts, while other studies focused on the use of digital mental health tools or psychotherapy outcomes. Importantly, these studies did not use a common measure of quality of life. Therefore, the available direct evidence does not allow a clear conclusion regarding the effect of mindfulness on quality of life among unemployed or economically inactive populations [24,25,26,27,28].

### 3.3. Evidence on Mindfulness and Sense of Coherence

The second search strategy identified studies examining mindfulness, meditation or related practices in relation to sense of coherence (SOC). The included studies encompassed diverse population and several were conducted in populations unrelated to unemployment. Accordingly, these findings were considered indirect evidence of the potential relationship between mindfulness and SOC, with limited direct applicability to unemployed populations.

Across intervention studies, the findings were generally favorable but not entirely consistent. Banaś-Ząbczyk et al. reported an increase in SOC and its three components among participants practicing Sudarshan Kriya Yoga compared with the comparison group [29]. Towsyfyan et al. also reported significant effects on SOC following an eight-week mindfulness-based program in women with breast cancer [30]. Gu et al. reported improvement in SOC following an online mindfulness-based stress reduction intervention among patients with cervical cancer [31].

In contrast, Spaccapanico Proietti et al. evaluated an eight-week mindful movement program among university students and found significant improvements in several domains, including positive mental health and some dimensions of interoceptive awareness, but the overall SOC did not improve significantly [32].

Observational studies provided additional evidence concerning the relationship between mindfulness and SOC. Sun et al. examined these constructs longitudinally in college students and found evidence supporting a mediating role of SOC in the relationship between mindfulness and subsequent psychological adjustment [33]. Uzdil and Günaydin reported a positive association between SOC and mindful attention among nursing students [34], whereas Bös et al. examined SOC and psychological characteristics in participants practicing yoga or pilates [35].

Schmitt et al. used a qualitative approach involving 17 individual interviews and one focus group discussion with employees from two organizations in the Czech Republic who completed an eight-week mindfulness-based intervention. The thematic analysis identified the intervention as providing resources including stress-reducing techniques, social support, a sense of coherence and a positive focus [36].

Taken together, the included studies provide heterogeneous but generally supportive evidence concerning mindfulness-related practices and SOC, although not all studies directly examined this relationship. However, the evidence combines randomized and non-randomized interventions with cross-sectional and longitudinal observational designs, and the populations differed substantially. Some studies also addressed related psychological or behavioral outcomes rather than SOC directly, further limiting comparability across studies. Therefore, these findings support a potential relationship between mindfulness and SOC but cannot be directly generalized to unemployed or economically inactive populations [29,30,31,32,33,34,35,36].

### 3.4. Characteristics of Mindfulness-Based and Related Interventions

The interventions included in the review varied substantially in terms of duration, intensity, frequency, content and delivery format. Duration ranged from a brief 15 min mindfulness meditation session [37] to multi-week programs, including a 4-week low-dose mindfulness-based intervention with weekly 1 h sessions [24], an 8-week mindful movement program comprising eight intensive group sessions with individual homework [32], and an 8-week mindfulness-based cognitive therapy program delivered through weekly sessions [30,36].

Delivery formats also differed, with Gu et al. evaluating an online MBSR program involving weekly digital materials and online follow-up [31]. Schmitt et al. described an eight-week synchronously online group mindfulness intervention with weekly sessions of two hours. The intervention included mindfulness training, supportive group discussion and theoretical content led by trained and certified facilitators [36].

Moreover, not all studies evaluated standardized mindfulness-based interventions. Related approaches included mindful movement [32], Sudarshan Kriya Yoga [29] and stress management training [27].

Overall, the heterogeneity in intervention duration, intensity, content and delivery format limits direct comparison between studies and prevents identification of an optimal intervention duration or intensity based on the included evidence.

### 3.5. Risk of Bias of the Included Studies

Risk of bias was assessed using the Joanna Briggs Institute (JBI) critical appraisal tool appropriate to each study design. As JBI does not establish universal thresholds for overall risk-of-bias categories, the overall judgments show in Appendix B represent a reviewer-based synthesis of the individual appraisal items.

Overall, methodological quality was heterogeneous. Most empirical studies were judged to have moderate risk of bias, while three quasi-experimental studies were considered to have high risk of bias. The main concerns were limited blinding, non-randomized allocation, incomplete reporting or attrition, limited control of confounding and, in some trials, the use of completer rather than intention-to-treat analyses.

These findings were considered when interpreting the evidence. In particular, the methodological limitations and heterogeneity of the included studies, together with the limited number of studies directly conducted in unemployed populations, limit the certainty with which the effects of mindfulness-related interventions on quality of life or SOC among unemployed adults can be inferred.

## 4. Discussion

### 4.1. Effects of Mindfulness on Quality of Life in Unemployed or Inactive Populations

The main aim of this review was to synthesize the existing evidence on the effects of mindfulness interventions on quality of life in unemployed or inactive populations. Direct evidence was limited and heterogeneous and the studies conducted in unemployed populations mainly addressing psychological distress, mental health or related outcomes rather than quality of life specifically [24,25,26,27,28]. Therefore, the available evidence does not allow firm conclusions to be drawn regarding the effectiveness of mindfulness interventions for improving quality of life in unemployed or inactive populations.

This finding should be interpreted within the broader context of interventions targeting unemployed people. Previous research has predominantly focused on employability, job-search skills and labor market reintegration, whereas fewer interventions have addressed health and well-being as primary outcomes [2,3,4,5]. Some programs combine health promotion, including nutrition, physical activity or stress-management strategies, with employment-related interventions, while others focus specifically on health promotion. A meta-analysis of health-oriented interventions among unemployed people found beneficial but generally limited effects, with considerable variability between interventions and outcomes [5]. These findings support the relevance of health promotion in this population, while also highlighting the difficulty of determining the effectiveness of specific intervention approaches.

Indirect evidence from other populations provides additional support for the potential relevance of mindfulness to quality of life. The included studies conducted in populations such as patients with cancer and university students reported improvements in some psychological or well-being outcomes [29,30,31,32]. However, these findings cannot be directly extrapolated to unemployed or inactive populations because of differences in population characteristics, health status and intervention contexts.

Another relevant issue is the duration of unemployment itself. Although evidence from studies published before the period covered by this review has associated longer periods of unemployment have been associated with poorer health outcomes [38], unemployment duration was not consistently reported in the interventions identified in this review. This limits the possibility of determining whether the potential effects of mindfulness-related interventions vary according to the length of time participants have been unemployed.

### 4.2. Mindfulness-Related Practices and Sense of Coherence

The second objective was to describe the available evidence concerning the relationship between mindfulness-related practices and sense of coherence (SOC). Although studies that evaluate mindfulness or meditation in other population groups are more numerous and consistently show reductions in anxiety, stress, depression and pain, as well as improved quality of life [15,16,17], the evidence concerning their relationship with SOC is less consistent. The included studies did not provide sufficient direct evidence to establish this relationship in unemployed or inactive populations; instead, most of the available evidence was indirect and derived from other populations.

Several intervention studies reported favorable changes in SOC or related salutogenic outcomes. Improvements in SOC were observed following Sudarshan Kriya Yoga, mindfulness-based programs in women with breast cancer and an online mindfulness-based stress reduction intervention in patients with cervical cancer [29,30,31]. Other studies examining conscious breathing or related practices have also reported favorable findings regarding SOC and self-compassion [29].

In contrast, the mindful-movement intervention among university students did not significantly improve overall SOC [32]. Observational studies additionally reported associations between mindfulness and SOC and suggested a potential mediating role of SOC in psychological adjustment [33,34,35]. The qualitative findings reported by Schmitt et al. (2025) [36] provide a complementary perspective, suggesting that mindfulness may contribute to the resources associated with a sense of coherence. However, because these findings were obtained qualitatively and in an employed population, they should be interpreted as complementary evidence rather than as evidence of an effect on SOC in unemployed or inactive populations.

Overall, the available evidence suggests a potential relationship between mindfulness-related practices and improvements in SOC and other salutogenic outcomes. However, the findings remain heterogeneous and predominantly indirect, and differences in populations, intervention characteristics and study designs, together with the limited evidence from unemployed or inactive populations, preclude a definitive conclusion regarding their effect on SOC in this population. These findings therefore support further investigation rather than a definitive causal conclusion.

### 4.3. Methodological Characteristics of the Interventions

Regarding the secondary objective, the interventions varied considerably in duration, content and delivery format.

Four-week modified programs were used by unemployed participants [24,26]. Across the broader body of included evidence, longer programs were also identified [30,32,36]. There are also differences in the delivery formats: some reviews incorporated mobile tools exclusively [25,26], others specified face-to-face sessions alongside these [24]. This predominance of digital formats may facilitate access to interventions, but it also limits opportunities to examine the potential contribution of interpersonal interaction within group-based health education.

Only a minority of studies targeting unemployed populations included pre- and post-intervention assessments, and none provided medium- or long-term follow-up [24,26]. Consequently, the durability of potential benefits remains uncertain. Furthermore, some studies reported the involvement of professionals with specific mindfulness expertise, whereas this information was not consistently described across studies [24,28].

The methodological heterogeneity identified is consistent with the broader literature on interventions for unemployed populations, in which differences in intervention characteristics and methodological quality make comparisons difficult [5]. This heterogeneity, together with the limited direct evidence, makes it difficult to determine whether differences in duration, delivery format or intervention characteristics contribute to differences in outcomes.

### 4.4. Implications for Future Research

Taken together, the findings indicate that mindfulness-related interventions may have potential as a health promotion approach, but the current evidence is insufficient to establish their effectiveness for improving quality of life or SOC specifically among unemployed or inactive populations.

Future studies should therefore specifically recruit these populations, clearly define intervention characteristics and outcomes, consider relevant contextual variables such as unemployment duration, and incorporate appropriate follow-up periods. This would help determine whether the favorable findings observed in other populations can be reproduced in people experiencing unemployment or economic inactivity.

## 5. Conclusions

This review identified limited and heterogeneous evidence regarding the effects of mindfulness-based interventions on quality of life in unemployed or inactive populations. The direct evidence available in unemployed populations was insufficient to establish a clear effect on quality of life, highlighting an important gap in the current literature.

Regarding the relationship between mindfulness-related practices and sense of coherence (SOC), the included studies provided predominantly indirect evidence from populations other than unemployed or inactive individuals. Although several studies reported favorable changes or associations between mindfulness-related practices and SOC, the heterogeneity of populations and study designs prevents definitive conclusions regarding a causal relationship or its applicability to unemployed populations.

The interventions identified among unemployed participants varied in duration, content and delivery format, with mobile-based approaches being common. The limited use of pre–post assessments and the absence of medium- or long-term follow-up in unemployed populations also limit conclusions regarding the persistence of potential effects.

Overall, the findings support the potential relevance of mindfulness-related interventions as a health-promotion approach, but do not provide sufficient evidence to establish their effectiveness for improving quality of life or SOC among unemployed or inactive populations. Further well-designed studies specifically targeting these populations are needed, with clearly described interventions, validated outcome measures, appropriate comparison groups and longer-term follow-up.

### Limitations

Analyzing the scientific evidence between 2021 and 2025 involves reviewing articles published during the COVID-19 pandemic, when some mindfulness and meditation-based interventions were adapted to digital and online formats. This context may limit the generalizability of the findings to other settings.

The use of mobile applications and online formats may reduce opportunities for face-to-face interaction and group-based health education.

Cultural differences and differences in age and sociodemographic characteristics require tailoring interventions to the population to ensure and improve their accessibility.

Individual traits may influence change, but their variability makes them difficult to assess consistently across studies.

Most studies do not record the duration of unemployment, making it difficult to analyze whether there is a relationship between the intervention effects and unemployment duration.

The restriction to studies published in English or Spanish may have introduced language bias and may have resulted in the exclusion of relevant evidence published in other languages.

The variability in populations, sample sizes, attrition, measurement instruments and study designs, as well as the lack of information in some articles, limits the comparability and generalizability of the findings. The methodological heterogeneity and risk of bias identified across the included studies limit the strength of the evidence and the certainty with which the effects of mindfulness-based interventions can be inferred for unemployed populations. No formal assessment of the certainty of the evidence was conducted, which limits the strength of the conclusions.

Finally, the second search strategy did not require an unemployed population, resulting in indirect evidence from other populations. Therefore, these findings cannot be directly generalized to unemployed adults.

In all cases, there is a lack of articles assessing interventions aimed at improving sense of coherence, quality of life and overall physical and mental health specifically among unemployed people. Therefore, the available evidence is insufficient to establish the effectiveness of mindfulness-based interventions in this population.

## Data Availability

No new data were created or analyzed in this study. Data sharing is not applicable to this article.

## Supplementary Materials

The following supporting information can be downloaded at https://www.mdpi.com/article/10.3390/healthcare14183024/s1, PRISMA 2020 flow diagram [39].

## Abbreviations

The following abbreviations are used in this manuscript:

GRRs	General Resources of Resistance
SOC	Sense of Coherence

## Appendix A

Highlighted information on each article, based on the type of study, objectives, study population and location, as well as the results obtained.

**Study**	**Country**	**Study Design**	**Population and Sample**	**Sampling/Recruitment**	**Intervention/Exposure**	**Duration/Follow-Up**	**Assessment Measures**	**Aims**	**Results**
Roemer et al. [24]	New Zealand	Quasi-experimental intervention study	Young unemployed adults; 239 recruited, 180 analyzed	Voluntary participation	Low-dose mindfulness-based intervention	4 weeks; pre- and post-intervention	Measures of mindfulness, well-being and psychological distress	To examine the effects of a low-dose mindfulness intervention on psychological distress in young unemployed adults	Mindfulness increased and psychological distress decreased; effects appeared greater among participants with higher baseline mindfulness and/or well-being.
Mata-Greve et al. [25]	USA	Analytical cross-sectional study	Adults who were unemployed due to COVID-19 or essential workers; 2485 recruited, 1987 analyzed	Voluntary online participation through Prolific	Use and perceived usability of digital mental health tools	Single assessment	PHQ-2, GAD-2, SBQ-R, CAGE-AID, System Usability Scale and Use Burden Scale	To examine psychological distress, use, usability and perceived characteristics of digital mental health tools	Digital tools were used by a minority of participants; meditation/mindfulness and information/education were among the preferred features.
Comtois et al. [26]	USA	Randomized clinical trial	Unemployed and essential workers; 3486 volunteers, 1356 randomized and 643 completed	Voluntary online recruitment through Prolific	Four commercial mobile mental health applications	4 weeks; baseline and post-intervention	PHQ-9, GAD-7, DERS-SF, SBQ-R, usability, acceptability and appropriateness measures	To examine effects, usability, acceptability and dose–response of mobile mental health applications	Depression and anxiety improved over time; no significant changes were found for suicidal behavior or emotional regulation.
Marasigan [27]	Philippines	Quasi-experimental pretest–posttest study with control group	Individuals who experienced job loss during the COVID-19 pandemic; 40 selected, 33 completed	NR	Stress-management skills training	Pre- and post-intervention	Ryff Psychological Well-Being Scale	To determine the effect of stress-management training on psychological well-being after job loss	Psychological well-being improved following the intervention.
Jiwani et al. [28]	Canada	Observational longitudinal study	Psychotherapy patients; 27,258 patients and 115,936 sessions	Naturalistic clinical sample	Employment status in relation to psychotherapy	Course of psychotherapy	Outcome Questionnaire-45 and clinical/engagement variables	To examine associations between employment status, baseline symptoms, engagement and psychotherapy outcomes	Unemployed patients showed higher baseline symptoms and suicide risk, but similar improvement trajectories to full-time workers.
Banaś-Ząbczy et al. [29]	USA	Quasi-experimental comparative study	Participants attending the YES!+ program; 260 participants	NR	Breathing-based meditation (Sudarshan Kriya Yoga)	Pre- and post-course assessment	SOC-29	To assess the influence of Sudarshan Kriya Yoga on sense of coherence	Statistically significant changes in SOC were reported following the breathing-based meditation program.
Towsyfyan et al. [30]	Iran	Quasi-experimental pretest–posttest study with control group	Women with breast cancer; the article reports 30 participants in the study groups	Convenience/purposive recruitment; random assignment to groups	Mindfulness-based cognitive therapy	8 weeks; 3-month follow-up	SOC-13, STAI and Emotional Self-Regulation Scale	To investigate the effects of mindfulness training on emotional self-regulation, anxiety and SOC	Mindfulness training significantly improved emotional self-regulation, reduced anxiety and increased SOC.
Gu et al. [31]	China	Randomized controlled trial	Women with cervical cancer; 102 eligible, 78 participated/completed	NR	Online mindfulness-based stress reduction	8 weeks; 3- and 6-month follow-up	Cancer-related fatigue, illness uncertainty, coping style, SOC and perceived social support measures	To examine the effects of online MBSR on psychological and disease-related outcomes	Improvements were observed in SOC and other psychological outcomes; several benefits were maintained at follow-up, although some effects were short-term.
Spaccapanico Proietti et al. [32]	Italy	Experimental pre–post pilot study without control group	Kinesiology and Sport Sciences students; 40 planned, 38 participated	Voluntary, first-come-first-served recruitment	Movimento Biologico mindful-movement program	8 weeks; pre- and post-intervention	PWB, PMH, SOC-13 and MAIA	To evaluate the short-term effects of mindful movement on psychological well-being, positive mental health, SOC and interoceptive awareness	Positive mental health and several interoceptive-awareness dimensions improved significantly; SOC and overall psychological well-being did not improve significantly.
Sun et al. [33]	Hong Kong, China	Longitudinal observational study	Public university students; 1627 at baseline and 253 at the second assessment	University-based recruitment; NR	Mindfulness and hope	Two-wave longitudinal assessment	Mindfulness, hope, SOC and internalizing/externalizing behaviors	To examine associations between mindfulness, hope, SOC and adjustment during COVID-19	Mindfulness was positively associated with SOC, and SOC mediated associations between mindfulness and subsequent adjustment.
Uzdil & Günaydın [34]	Turkey	Analytical cross-sectional study	Nursing students; 410 participants	Voluntary online participation	Mindful attention awareness in relation to SOC	Single assessment	SOC-13, MAAS and Academic Self-Efficacy Scale	To determine the relationship between SOC, mindful attention awareness and academic self-efficacy	SOC was moderately positively related to mindful attention awareness; SOC and mindful attention awareness explained 36.4% of the variance in SOC.
Bös et al. [35]	Germany, Austria and Switzerland	Analytical cross-sectional study	Yoga and pilates practitioners; N = 246	NR	Yoga involvement compared with pilates	Single assessment	SOC-L9, WHO-5, MI-RSWB and BSI-18	To examine relationships between yoga involvement, psychological well-being, SOC and psychological distress	Yoga involvement was associated with higher SOC and spiritual well-being; involvement mediated the relationship between practice and spiritual well-being.
Schmitt et al. [36]	Czech Republic	Qualitative study	Employees from two corporate organizations: an IT company (~150 employees) and a marketing company (~250 employees). In total, 96 people participated in the mindfulness intervention; qualitative data comprised 17 individual interviews and one focus group with 5 participants.	Purposive sampling for qualitative participants. Recruitment for the intervention was conducted by email through HR representatives.	Eight-week online group mindfulness course based on principles and techniques of Mindfulness-Based Cognitive Therapy, including mindfulness training, supportive group discussion and theory. Sessions covered mindfulness, present-moment awareness, breathing, reactivity, kindness/compassion, thoughts and continued practice.	8 weeks; weekly 2 h sessions. Qualitative interviews were conducted shortly after the intervention. No longer-term follow-up reported.	Semi-structured individual interviews (~45 min) and one focus group (90 min). Thematic analysis was conducted using the Job Demands–Resources framework.	To explore how a mindfulness intervention aimed at promoting well-being and protecting mental health supports employees during organizational uncertainty associated with M&A and the aftermath of the pandemic.	Nine themes were identified, including high workload, stress, work–home conflict, stress-reducing techniques, social support, sense of coherence, positive focus, exploring new experiences, and the intervention itself as a demand.
Somaraju et al. [37]	Online; USA, Australia and India	Randomized experimental study	55 non-meditators	Random assignment	Brief mindfulness meditation induction	Single 15 min session	Trait mindfulness, mind-wandering, positive and negative affect and Sustained Attention to Response Task measures	To investigate the effects of a brief mindfulness meditation induction	A single 15 min induction did not produce statistically significant changes in mindfulness or negative affect compared with control conditions.
NR = not reported. MBSR, mindfulness-based stress reduction; SOC, sense of coherence; SOC-13/SOC-29, 13-/29-item sense of coherence scale; MAAS, Mindful Attention Awareness Scale; PHQ-2/PHQ-9, Patient Health Questionnaire; GAD-2/GAD-7, Generalized Anxiety Disorder Scale; DERS-SF, Difficulties in Emotion Regulation Scale—Short Form; SBQ-R, Suicide Behaviors Questionnaire—Revised; STAI, State-Trait Anxiety Inventory; PWB, psychological well-being; PMH, positive mental health; MAIA, Multidimensional Assessment of Interoceptive Awareness; WHO-5, WHO—Five Well-Being Index; MI-RSWB, Multidimensional Inventory for Religious/Spiritual Well-Being; BSI-18, Brief Symptom Inventory—18; BDI-II, Beck Depression Inventory—II.									

## Appendix B

**Table A1:** Risk of bias assessment of the included studies using the Joanna Briggs Institute (JBI) critical appraisal tools. The principal methodological concerns identified were incomplete follow-up, lack of blinding where applicable, non-randomized allocation, absence of a control group in the single-group intervention study, self-reported exposures, insufficient control of confounding in observational studies, and incomplete methodological reporting in some studies. These concerns were considered when interpreting the findings.

Study	Design	Yes	No	Unclear
Roemer et al. [24]	Quasi-experimental	8	1	0
Mata-Greve et al. [25]	Analytical cross-sectional	6	1	1
Comtois et al. [26]	RCT	9	1	1
Marasigan [27]	Quasi-experimental	6	2	1
Jiwani et al. [28]	Cohort	7	1	2
Banaś-Ząbczyk et al. [29]	Quasi-experimental	5	2	2
Towsyfyan et al. [30]	Quasi-experimental	8	0	1
Gu et al. [31]	RCT	9	2	1
Spaccapanico Proietti et al. [32]	Quasi-experimental	5	1	2
Sun et al. [33]	Cohort/longitudinal	5	1	2
Uzdil & Günaydın [34]	Analytical cross-sectional	7	0	1
Bös et al. [35]	Analytical cross-sectional	8	0	0
Schmitt et al. [36]	Qualitative study	8	0	2
Somaraju et al. [37]	RCT	7	4	1
